# Glucose Hypometabolic Pattern and Prediction of Isocitrate Dehydrogenase Status in Non-Contrast-Enhanced Glioma Using Z-Score-Based ^18^F-Fluorodeoxyglucose Positron Emission Tomography

**DOI:** 10.3390/cancers18142298

**Published:** 2026-07-17

**Authors:** Naoya Imai, Hirohito Yano, Yuka Ikegame, Shoji Yasuda, Ryo Morishima, Yoshinori Kumagai, Soko Ikuta, Takashi Maruyama, Naoyuki Ohe, Morio Kumagai, Yoshihiro Muragaki, Jun Shinoda, Tsuyoshi Izumo

**Affiliations:** 1 Department of Neurosurgery, Chubu Neurorehabilitation Hospital, Chubu Medical Center for Prolonged Traumatic Brain Dysfunction, Minokamo 505-8503, Japan; ikegame-nsu@umin.ac.jp (Y.I.); gumngng@icloud.com (R.M.); mor123155@yahoo.co.jp (M.K.); junshino@joy.ocn.ne.jp (J.S.); 2 Department of Neurosurgery, Gifu University Graduate School of Medicine, Gifu 501-1194, Japan; show_jis@yahoo.co.jp (S.Y.); yo.kuma13@gmail.com (Y.K.); ohe.naoyuki.r3@f.gifu-u.ac.jp (N.O.); go-izumo@hotmail.co.jp (T.I.); 3 Department of Clinical Brain Sciences, Gifu University Graduate School of Medicine, Gifu 501-1194, Japan; 4 Department of Neurosurgery, Tokyo Women’s Medical University, Tokyo 162-8666, Japan; sikuta@twmu.ac.jp (S.I.); mtm2727@gmail.com (T.M.); ymuragaki@twmu.ac.jp (Y.M.)

**Keywords:** ^18^F-fluorodeoxyglucose positron emission tomography, non-contrast-enhanced gliomas, glucose hypometabolic pattern, prediction, isocitrate dehydrogenase status

## Abstract

**Background/Objectives:** ^18^F-fluorodeoxyglucose positron emission tomography (FDG PET) reflects glucose metabolism but has limited diagnostic value for gliomas because of its high physiological uptake in the normal brain. This study visualized and characterized patterns of hypometabolism in non-contrast-enhanced gliomas using Z-score analysis and explored its potential for estimating isocitrate dehydrogenase (IDH) status. **Methods:** We retrospectively analyzed 116 patients with non-contrast-enhanced supratentorial gliomas, classified according to the 2021 World Health Organization classification (26 glioblastomas, IDH-wildtype; 54 astrocytomas, IDH-mutant; and 36 oligodendrogliomas, IDH-mutant and 1p/19q-codeleted). Z-score maps of FDG images were generated using a normal database. Tumor-associated hypometabolic regions (Z < −2) were analyzed, focusing on the proportion of severely hypometabolic regions (Z6–: Z < −6) and sphericity. **Results:** The mean Z-score differed significantly among the three subtypes, with the highest value observed in glioblastomas and the lowest in astrocytomas (*p* < 0.001). IDH-mutant gliomas showed a higher proportion of Z6– regions (*p* < 0.001) and higher sphericity (*p* = 0.009) than IDH-wildtype gliomas. In multivariable logistic regression, both the proportion of Z6– and sphericity were independent predictors of IDH status. Their combination demonstrated a strong discriminative performance for IDH status (area under the receiver operating characteristic curve = 0.839). On visual assessment, tumors with both small Z6– extent and low sphericity were primarily IDH-wildtype (75%), whereas the absence of both features indicated IDH-mutant status (94.8%). **Conclusions:** Z-score-based analysis enables the objective visualization and quantification of tumor-associated hypometabolic regions in non-contrast-enhanced gliomas, providing a potentially useful imaging biomarker for estimating IDH status.

## Simple Summary

Non-contrast-enhanced gliomas are often difficult to evaluate using ^18^F-fluorodeoxyglucose positron emission tomography (FDG PET) because they typically do not show increased FDG uptake. Instead of focusing on areas with increased uptake, we investigated areas with decreased uptake using Z-score-based analysis. This method enables the visualization and quantitative assessment of tumor-associated hypometabolic regions. We found that gliomas with isocitrate dehydrogenase (IDH) mutations tended to exhibit a higher proportion of severely hypometabolic regions and a more spherical hypometabolic pattern than IDH-wildtype gliomas. These findings suggest that the quantitative evaluation of tumor-associated hypometabolic regions on FDG PET may provide a useful imaging biomarker for estimating IDH status in patients with non-contrast-enhanced gliomas.

## 1. Introduction

^18^F-Fluorodeoxyglucose positron emission tomography (FDG PET) is a versatile imaging technique that reflects glucose metabolism. In neuro-oncology, FDG PET assists in the diagnosis and prognostic evaluation of primary central nervous system lymphomas, the identification of metastatic brain tumors, and evaluation of tumor recurrence after treatment [1,2,3].

For gliomas, FDG PET is used to assess tumor grade, predict prognosis [4], and differentiate glioblastomas [5]. However, the brain inherently exhibits high glucose metabolism, and normal brain tissue demonstrates intense FDG uptake [6]. Additionally, there are physiological variations in uptake between regions, such as the gray and white matter. Therefore, the visualization and quantitative assessment of tumor activity are challenging, limiting the clinical utility of FDG PET in gliomas [7]. Furthermore, low-grade gliomas exhibit lower FDG uptake than normal brain parenchyma, further restricting the diagnostic value of FDG PET [2].

Historically, the absence of contrast enhancement has often been considered suggestive of a lower-grade glioma. However, the 2021 World Health Organization (WHO) classification represents a paradigm shift toward molecular classification systems [8]. Gliomas with wildtype isocitrate dehydrogenase (IDH) may be classified as glioblastomas, IDH-wildtype, regardless of the presence or absence of contrast enhancement and other malignant radiological features [8]. Accordingly, IDH-wildtype gliomas lacking the typical imaging features of glioblastoma, such as contrast enhancement or central necrosis, are increasingly recognized as an important subgroup of gliomas [9,10]. Because glioblastoma and IDH-wildtype gliomas have poor prognoses, accurate preoperative imaging assessment is particularly important even in the absence of contrast enhancement [11]. However, diagnosis remains challenging in non-contrast-enhanced gliomas.

Therefore, we aimed to elucidate the differences in metabolic patterns among molecular subtypes, focusing on the glucose hypometabolic areas of tumors using Z-score mapping on FDG PET. The Z-score represents the degree of deviation of each voxel’s uptake from the mean and standard deviation (SD) of the normal control database. Using Z-score-based analysis, we characterized the FDG hypometabolic profiles of glioblastoma, IDH-wildtype (G); astrocytoma, IDH-mutant (A); and oligodendroglioma, IDH-mutant and 1p/19q-codeleted (O). We also examined whether the Z-score map could enable tumor visualization and IDH status prediction by stratifying gliomas according to low glucose metabolism.

Our analysis revealed distinct hypometabolic patterns in different glioma subtypes. These patterns were useful for identifying IDH-wildtype gliomas among non-contrast-enhanced gliomas.

## 2. Materials and Methods

### 2.1. Patients

This retrospective study included patients who underwent FDG PET and magnetic resonance imaging (MRI) on the same day at our institution between May 2012 and December 2022. During the study period, 527 patients with gliomas underwent preoperative PET and MRI. Pathological diagnoses were confirmed in 245 patients. We excluded 129 patients because of MRI contrast-enhanced lesions, infratentorial lesions, unknown genetic mutations, or age < 18 years. Therefore, 116 patients were included in the analysis.

The tumors were classified based on histopathological findings and available molecular data, including IDH immunohistochemistry and 1p/19q status, with reference to the 2021 WHO classification of central nervous system tumors. IDH mutation status was determined using immunohistochemical staining for IDH1 R132H and IDH2 R172 on formalin-fixed, paraffin-embedded tissue sections, as previously described [12]. Cases showing strong diffuse immunoreactivity in tumor cells were considered positive. The 1p/19q codeletion status was evaluated using fluorescence in situ hybridization.

This study was approved by the Institutional Ethics Review Board of Chubu Neurorehabilitation Hospital (No. 2025-08) and conducted in accordance with the Declaration of Helsinki. The requirement for informed consent was waived due to the retrospective design of the study. Patients could opt out after reviewing the documents disclosed at https://cnrh.jp/wp/wp-content/uploads/2025/05/optout20250508.pdf (accessed on 12 July 2026).

### 2.2. MRI Procedure

MRI was performed using an Achieva 3.0 Tesla TX QD MRI system (Philips, Amsterdam, The Netherlands) in all patients. T1-weighted imaging (T1WI) and T2-weighted imaging (T2WI) were performed using the following parameters: repetition times of 2100 and 2500 ms, echo times of 9.5 and 230 ms, flip angles of 90° and 90°, matrix sizes of 512 × 512 and 240 × 240, fields of view of 230 × 230 and 240 × 240 mm, and slice thickness of 5 mm. For contrast-enhanced imaging, 9 mL of a gadolinium-based contrast agent was administered intravenously, followed by a 20-mL saline flush.

### 2.3. PET Procedure

FDG PET imaging was performed using an Eminence STARGATE (Shimadzu Corporation, Kyoto, Japan) operating in three-dimensional (3D) acquisition mode. The scanner generated 99 transaxial slices with an interslice distance of 2.65 mm and an in-plane spatial resolution of 4.8 mm (full width at half maximum). Images were obtained parallel to the canthomeatal line. FDG tracers were administered at a dose of 3.5 MBq/kg after a fasting period of at least 5 h, and tracer uptake was quantified using the standardized uptake value (SUV). During the postinjection uptake period, the patients rested in the supine position in a quiet room with their eyes closed.

### 2.4. Analysis Technique

High-intensity areas on T2-weighted images were semiautomatically segmented three-dimensionally as the volume of interest (VOI)-T2 using Dr. View/Linux software (R2.5.0; AJS Corporation, Tokyo, Japan). After initial segmentation based on the T2 signal intensity, the lesions were manually adjusted when necessary. The entire T2-hyperintense lesion, including the peritumoral edema and cystic components, when present, was included as a VOI. In non-contrast-enhanced gliomas, tumor infiltration and peritumoral edema cannot be reliably distinguished using MRI. Therefore, the entire T2-hyperintense region was regarded as the tumor extent. The segmentations were reviewed by two board-certified neurosurgeons who were blinded to the study objectives and finalized by consensus.

**Figure 1:**
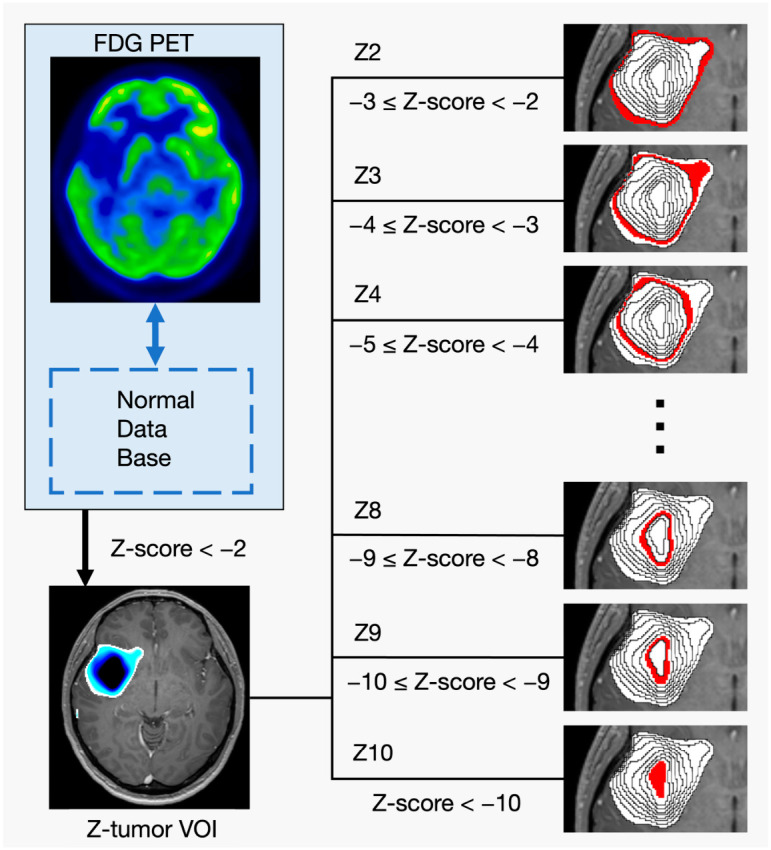
Workflow for Z-score-based ^18^F-fluorodeoxyglucose positron emission tomography (FDG PET) analysis and visualization of hypometabolic regions. Z-score maps were generated using a normal database to visualize hypometabolic regions in gliomas. Voxels with a Z-score < −2 were defined as tumor-associated hypometabolic regions (Z-tumor). Hypometabolic voxels were further categorized into ordinal ranges according to the severity of glucose hypometabolism. The corresponding volume of interest (VOI) for each Z-score interval is shown in red: Z2 (−3 ≤ Z < −2), Z3 (−4 ≤ Z < −3), Z4 (−5 ≤ Z < −4), Z5 (−6 ≤ Z < −5), Z6 (−7 ≤ Z < −6), Z7 (−8 ≤ Z < −7), Z8 (−9 ≤ Z < −8), Z9 (−10 ≤ Z < −9), and Z10 (Z < −10).

For FDG PET analysis, the tumor-to-normal (T/N) ratio was calculated using Dr. View/Linux software (R2.5.0; AJS Corporation, Tokyo, Japan). The T/N ratio was defined as the ratio of the tumor SUV to the mean SUV of the contralateral normal cortex. The maximum and minimum T/N ratios were calculated using the maximum and minimum SUVs within the tumor, respectively. Statistical Z-score maps were generated using the Easy Z-score Imaging System (eZIS; Fujifilm RI Pharma, Tokyo, Japan) [13]. eZIS combines anatomical standardization with SPM2 and voxel-wise Z-score mapping. Voxel-wise age-adjusted Z-scores, representing the number of SDs from the mean of age-matched normal controls, were calculated to quantify deviations from normal metabolism. Registration accuracy was visually confirmed using the FDG template and normalized T2-WIs, and no gross mismatch was observed. In addition to the normal database built into the eZIS program, we constructed a local database of 99 healthy individuals aged 20–60 years who underwent scans using the same acquisition and preparation conditions as those used for the study patients at our institution, with approval from the institutional ethics committee (No. 24-004). Voxels with a Z-score < −2 were considered hypometabolic, whereas those with a Z-score > 2 were considered hypermetabolic. The threshold of Z < −2 was predefined before the analysis because it represents a deviation of more than two SDs below the normal mean and is widely used to define abnormal hypometabolism in voxel-based brain PET analyses [14]. Because the primary aim of this study was to evaluate hypometabolism, the Z-tumor was defined as a hypometabolic region (Z < −2) associated with a tumor. For tumor analysis, tumor-associated hypometabolic regions were identified with reference to VOI-T2 and collectively defined as the Z-tumor VOI. If multiple or extensive hypometabolic regions were present, the tumor-associated portion was selected by visually excluding areas considered clearly unrelated to the tumor. The first author defined the Z-tumor VOIs, which were independently reviewed by a board-certified neurosurgeon blinded to the study objectives. To formally assess the interobserver reproducibility of Z-tumor delineation, a third board-certified neurosurgeon, who did not have access to the original segmentations, independently generated Z-tumor VOIs in a stratified random subset of 30 cases with a balanced representation of G, A, and O (10 each) using the same predefined criteria. Agreement between the original and repeated Z-tumor VOIs was evaluated using the Dice similarity coefficient. Z-scores within each Z-tumor were further categorized into ordinal ranges to quantify the severity of hypometabolism: Z2 (−3 ≤ Z < −2), Z3 (−4 ≤ Z < −3), Z4 (−5 ≤ Z < −4), Z5 (−6 ≤ Z < −5), Z6 (−7 ≤ Z < −6), Z7 (−8 ≤ Z < −7), Z8 (−9 ≤ Z < −8), Z9 (−10 ≤ Z < −9), and Z10 (Z < −10) (Figure 1). These ordinal categories were used to explore the distribution of tumor-associated hypometabolism. Candidate parameters for visual assessment were subsequently selected based on the observed distribution.

To enhance the visual assessment of tumor morphology on Z-score maps, we evaluated the morphological characteristics of Z-tumors by calculating their sphericity using MATLAB R2022b (MathWorks, Natick, MA, USA). Sphericity was defined as the ratio of the surface area of a sphere with the same volume as that of the lesion to the actual surface area of the lesion, with values closer to 1 indicating a more spherical shape. Lower sphericity values reflected more irregular or elongated tumor shapes. Sphericity has been used in MRI-based glioma studies to characterize tumor shape and infiltration patterns [15].

To facilitate clinical applicability, we aimed to identify imaging parameters for the visual assessment of IDH status. Hypometabolic distribution and sphericity patterns were selected as visually interpretable candidates. These variables were entered into a multivariate logistic regression analysis to evaluate their independent associations with IDH status. The parameters that remained significant in the multivariate analysis were subsequently used to construct the prediction models. The predictive performance of each parameter, individually and in combination, was compared using receiver operating characteristic (ROC) curve analysis.

### 2.5. Visual Validation Analysis

To assess the visual utility of the selected variables for IDH status, three neurosurgeons, including one board-certified in nuclear medicine, performed visual evaluations. Evaluations were based on the mean values of the IDH-wildtype and IDH-mutant groups and the ROC-based cutoff values for the selected parameters, which were divided into four ranges. Representative cases corresponding to the three reference values were presented as visual references, and each case was classified using a four-point visual rating scale. Interobserver agreement was assessed using pairwise-weighted Cohen’s kappa statistics. The median scores of the three neurosurgeons were used for the analysis. Correlations between visual rating scores and quantitative parameters were examined, and their relevance to IDH status was evaluated.

### 2.6. Data Analysis

Patient characteristics, T/N ratio, Z-score, VOI volume, and sphericity were compared across the glioma subtypes and IDH statuses. Categorical variables were analyzed using the Pearson chi-square test, whereas continuous variables were analyzed using the Mann–Whitney *U* or Kruskal–Wallis tests, as appropriate. The Steel–Dwass test was used for multiple comparisons. A paired *t*-test was used to compare the VOI within the same patient. ROC analysis was performed to determine the optimal cutoff values, sensitivity, specificity, and area under the ROC curve (AUC). The optimal cutoff values were determined using the Youden index. The differences between ROC curves were assessed using DeLong’s test. DeLong’s test and pairwise-weighted Cohen’s kappa analyses were performed using EZR software (v.R4.5.0; Saitama Medical Center, Jichi Medical University, Saitama, Japan; https://www.jichi.ac.jp/usr/hema/EZR/statmed.html; accessed on 12 July 2026). All other statistical analyses were performed using JMP statistical software (v.17.1.0; SAS Institute Inc., Cary, NC, USA). Statistical significance was set at *p* < 0.05.

Because the normal database of the eZIS program includes people aged ≤ 69 years, a sensitivity analysis was performed after excluding patients aged ≥ 70 years to confirm that the diagnostic performance for IDH status remained similar.

## 3. Results

### 3.1. Patient Characteristics

The clinical characteristics of the 116 patients are summarized in Table 1. Of these, 26 were classified as G, 54 as A, and 36 as O. The G group was significantly older than the A and O groups (*p* < 0.001). No significant difference in the VOI-T2 volume was observed among the three subtypes (*p* = 0.32). Pseudopalisading necrosis was not observed.

### 3.2. FDG Metabolic Patterns

**Figure 2:**
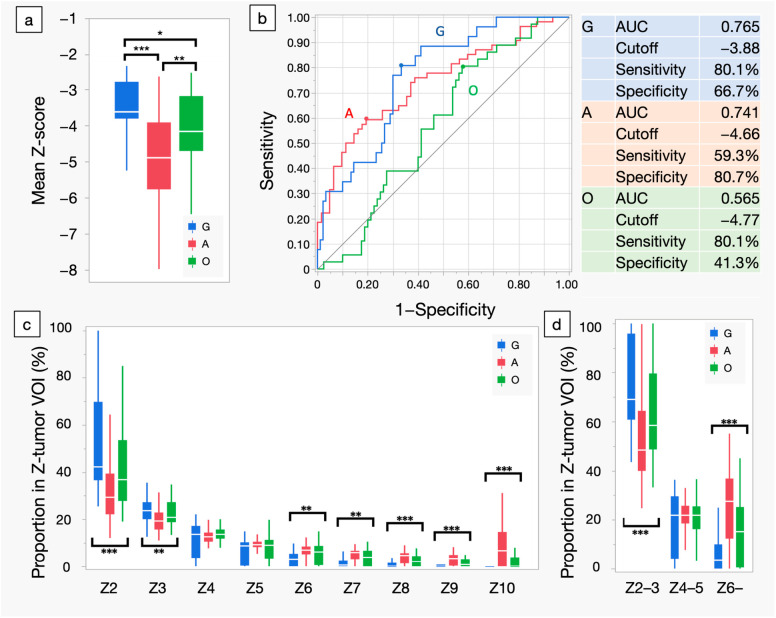
Z-score-based ^18^F-fluorodeoxyglucose positron emission tomography characteristics of the glioma subtypes. (**a**) Mean Z-scores within the Z-tumor volume of interest (VOI) for glioblastoma, isocitrate dehydrogenase (IDH)-wildtype (G), astrocytoma, IDH-mutant (A), and oligodendroglioma, IDH-mutant, and 1p/19q-codeleted (O). Lower mean Z-scores indicate more severe tumor-associated hypometabolism. (**b**) Receiver operating characteristic curves for distinguishing each subtype from the others using mean Z-scores. The area under the curve (AUC), optimal cutoff, sensitivity, specificity, and Youden index are shown. (**c**) Proportional distribution of Z-score ranges within the Z-tumor VOI for each subtype. (**d**) Z-score categories grouped into mildly decreased (Z2–Z3), intermediate (Z4–Z5), and severely decreased (Z6–) hypometabolic regions for exploratory comparison among the different glioma subtypes. Statistical significance: * *p* < 0.05, ** *p* < 0.01, *** *p* < 0.001.

The maximum and minimum T/N ratios, as well as the mean and minimum Z-scores of the Z-tumors, were significantly lower in the A group than in the other groups (all *p* < 0.001). Among these parameters, only the mean Z-score showed significant differences across all pairwise comparisons, with the lowest value in the A group and the highest in the G group (*p* < 0.001) (Figure 2a, Table 1). The ROC curves for discriminating each glioma subtype from the other two subtypes are shown in Figure 2b. In Z-tumors, the proportions of Z2 and Z3 were ordered as A < O < G, indicating that these hypometabolic ranges were more extensive in the G group. The differences between the G, A, and O groups disappeared at Z4 and Z5. From Z6 to Z10, the order was reversed (A > O > G), indicating that the more severely hypometabolic areas were larger in the A group (Figure 2c). To facilitate visual interpretation, the Z-score ranges were grouped according to their characteristic distribution patterns among the three glioma subtypes. The parameters were divided into three categories: Z2–3 (Z2 and Z3) regions that were wider in the G group, Z4–5 (Z4 and Z5) regions that showed no significant difference among the groups, and Z6– (Z-score < −6) regions that were wider in the A group. The proportions of these three categories are shown in Figure 2d. Hypermetabolic regions (Z > 2) were observed in only three cases (one in G and two in O), and all these cases also involved coexisting hypometabolic regions.

**Figure 3:**
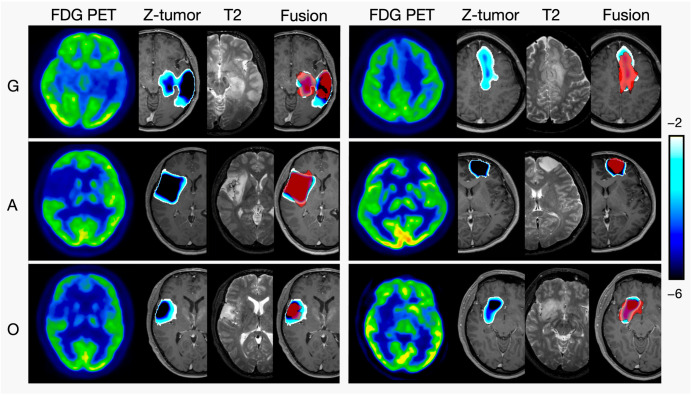
Representative cases showing ^18^F-fluorodeoxyglucose positron emission tomography (FDG PET), Z-tumor volume of interest (VOI), and T2-weighted images. Representative cases are shown for glioblastoma, isocitrate dehydrogenase (IDH)-wildtype (G), astrocytoma, IDH-mutant (A), and oligodendroglioma, IDH-mutant and 1p/19q-codeleted (O). The Z-tumor VOI was defined as a tumor-associated hypometabolic region with Z-scores < −2. Z-tumor VOI was fused with the T1-weighted image, and the color scale represents Z-scores from −6 to −2. The fusion image shows the VOI-T2 (red), corresponding to the T2-hyperintense region, overlaid on the Z-tumor VOI.

The sphericity of the Z-tumor was significantly lower in the G group than in the A and O groups (*p* = 0.009) (Table 1). Representative cases of G, A, and O are shown in Figure 3. In these images, Z-scores ranging from −6 to −2 were applied to improve the visual contrast and make the differences easier to interpret. Within the Z-tumor, representative G cases showed less extensive severe hypometabolic regions (dark-colored areas) and a more irregular Z-tumor shape than the IDH-mutant subtypes (Figure 3).

### 3.3. Interobserver Reproducibility of Z-Tumor Delineation

The interobserver reproducibility of Z-tumor delineation was evaluated in a stratified random subset of 30 cases. The Dice similarity coefficient between the original and independently re-delineated Z-tumor VOIs had a median value of 0.998 (interquartile range, 0.971–1.000).

### 3.4. IDH Prediction

**Figure 4:**
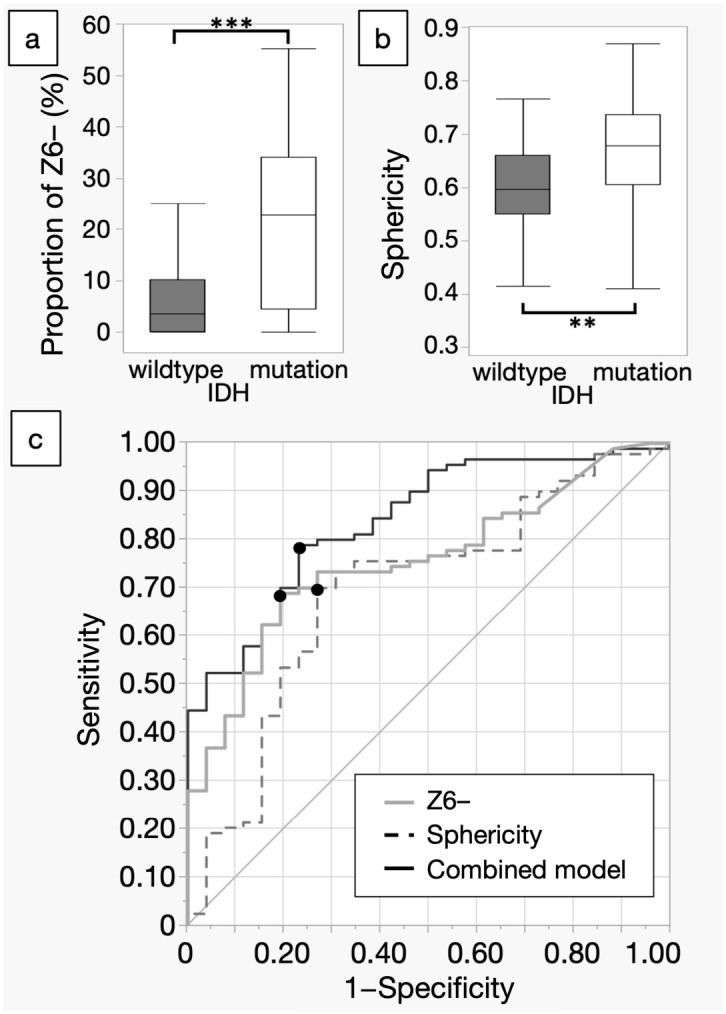
Proportion of severe hypometabolic regions (Z6–), sphericity, and their diagnostic performance for predicting isocitrate dehydrogenase (IDH) status. (**a**) Boxplots of Z6– values according to IDH status. (**b**) Boxplots of sphericity values according to IDH status. (**c**) Receiver operating characteristic curves for predicting IDH status using Z6– (gray), sphericity (dashed), and the combined model incorporating Z6– and sphericity (black). The area under the curve of the combined model (0.839) was significantly higher than that of Z6– (0.755, *p* = 0.012) and sphericity (0.694, *p* = 0.001). ** *p* < 0.01, *** *p* < 0.001.

Based on the observed distribution of hypometabolic regions across the ordinal Z-score categories, Z6– (Z-score < −6) was selected as an exploratory threshold for subsequent visual assessment because no established threshold exists for severe tumor-associated hypometabolism. This proportion was markedly larger in IDH-mutant gliomas (21.9 ± 16.6%) than in IDH-wildtype gliomas (7.1 ± 9.5%, *p* < 0.001) (Figure 4a). We also assessed tumor morphology using sphericity as a second visually interpretable parameter. Sphericity was lower in IDH-wildtype gliomas (0.6 ± 0.11) than in IDH-mutant gliomas (0.67 ± 0.1, *p* = 0.003) (Figure 4b). In the multivariable logistic regression analysis, the proportion of Z6– regions and sphericity were independently and significantly associated with IDH-mutant status (Z6–: *p* < 0.001, sphericity: *p* = 0.002) (Supplementary Material S1). To assess their discriminative performance, we evaluated three models: Z6 alone, sphericity alone, and a combination of both. The combined model demonstrated a higher AUC (0.839) than each predictor alone (Z6–: 0.755, *p* = 0.012; sphericity: 0.694, *p* = 0.001) (Figure 4c; Supplementary Material S2).

### 3.5. Visual Validation

**Figure 5:**
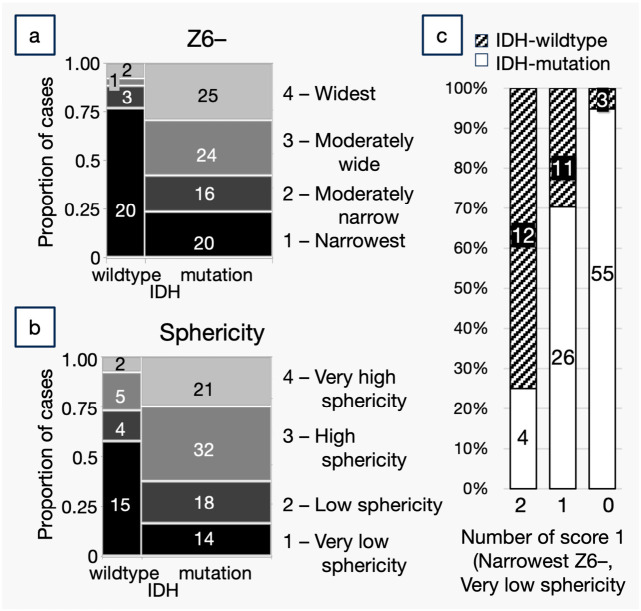
Visual classification of the proportion of severe hypometabolic regions (Z6–) and sphericity according to IDH status. (**a**) Four-grade visual classification of the proportion of Z6–, ranging from the narrowest (score 1) to the widest (score 4). (**b**) Four-grade visual classification of the sphericity, ranging from very low (score 1) to very high (score 4). (**c**) Proportion of isocitrate dehydrogenase (IDH)-mutant and IDH-wildtype gliomas according to the number of parameters rated as 1 (very low sphericity and narrowest Z6– extent). Numbers within the bars indicate the number of patients.

Z6– and sphericity were independently associated with IDH status; therefore, they were selected for visual validation. The validation study classified each parameter into four visual grades based on their quantitative distributions: (1) below the IDH-wildtype mean, (2) between the IDH-wildtype mean and the ROC-based cutoff value, (3) between the ROC-based cutoff value and the IDH-mutant mean, and (4) above the IDH-mutant mean. These were qualitatively labeled as narrowest to widest (for Z6– extent) and least to most spherical (for sphericity) (Supplementary Material S3a,b). Five representative cases are shown for illustration, and the remaining 111 cases (26 IDH-wildtype and 85 IDH-mutant) were analyzed. Significant positive correlations were found between the visual scores and quantitative values for both Z6– (ρ = 0.86, *p* < 0.001) and sphericity (ρ = 0.67, *p* < 0.001) (Supplementary Material S3c). IDH-wildtype cases were significantly more frequent among those with a visual score of 1 for both Z6– (*p* < 0.001, Figure 5a) and sphericity (*p* < 0.001, Figure 5b). These visual features were subsequently combined to evaluate their utility in predicting IDH status (Figure 5c). When both parameters had a score of 1, 75% of the cases were IDH-wildtype; when one parameter had a score of 1, 70.3% were IDH-mutant; and when neither parameter had a score of 1, 94.8% were IDH-mutant (Figure 5c).

The interobserver agreement for the four-point visual ratings was subsequently evaluated. The pairwise-weighted Cohen’s kappa values ranged from 0.561 to 0.731 (median, 0.646) for sphericity and from 0.810 to 0.850 (median, 0.844) for the Z6– extent.

### 3.6. Volumetric Analysis

The volume of the Z-tumor VOI (Z < −2) was 57.1 ± 52.9 mL, which was significantly larger than that of VOI-T2 (33.7 ± 30.2 mL, *p* < 0.001). At Z < −3 (39.3 ± 40.8 mL), the volume remained significantly larger than that of VOI-T2 (*p* = 0.006), whereas at Z < −4 (28.2 ± 32.3 mL), it became significantly smaller (*p* = 0.001) than that of VOI-T2 (Supplementary Material S4).

### 3.7. Sensitivity Analysis

In a sensitivity analysis excluding the three patients aged ≥ 70 years who were not represented in the normal database (71, 77, and 89 years; all in the G group), 113 patients were analyzed. The proportion of Z6– was significantly lower in the IDH-wildtype than in the IDH-mutant groups (7.6% vs. 21.9%, *p* < 0.001; AUC, 0.745). Sphericity was also significantly lower in the IDH-wildtype than in the IDH-mutant groups (0.60 vs. 0.67, *p* = 0.003; AUC, 0.699). In the multivariable model, both Z6– (*p* < 0.001) and sphericity (*p* = 0.004) remained independent predictors of IDH status, and the combined model yielded an AUC of 0.832.

## 4. Discussion

Our Z-score-based analysis of metabolic decreases in gliomas provides a clear visualization and quantitative evaluation of tumor-associated hypometabolism. The mean Z-score was lower in the A group and higher in the G group. In the G group, relatively mild hypometabolic regions were more widespread, whereas in the A group, more severe hypometabolic areas were predominant. These intergroup differences were not detected using the T/N ratio but were revealed through the Z-score analysis.

Previous FDG PET studies on gliomas that relied on T/N ratios or visual assessments have shown limited diagnostic utility [2,7]. Conventionally, higher-grade gliomas often demonstrate increased FDG uptake. However, in our cohort restricted to non-contrast-enhanced gliomas, hypermetabolic regions (Z > 2) were observed in only three cases, whereas most tumors predominantly showed hypometabolism. Therefore, our hypometabolism-based approach should be regarded as complementary to conventional FDG PET assessment, particularly in non-contrast-enhanced gliomas without overt hypermetabolic features. Z-score analysis provides objective, quantitative, and reproducible evaluations by reducing observer-dependent variability [16,17]. It also enhances visual interpretability, making subtle metabolic changes easier to observe. The use of a predefined threshold (Z < −2) allowed Z-tumor delineation to be performed in a relatively rule-based manner. Consistently, Z-tumor delineation showed a high Dice similarity coefficient in the subset analysis of 30 cases, indicating good interobserver reproducibility. Although Z-score analysis has been widely used to evaluate dementia and brain function, it has not been applied to the assessment of glioma.

One possible reason for hypometabolism is the loss of normal brain cells with high glucose metabolism. Tumor-induced compression or infiltration reduces the density of normal brain cells. IDH-mutant gliomas tend to have a more circumscribed morphology than IDH-wildtype gliomas [18,19]. Accordingly, pronounced hypometabolism likely reflects the compression and displacement of surrounding normal cells. In contrast, IDH-wildtype gliomas may show a more infiltrative growth pattern, allowing the coexistence of normal brain cells within the tumor. While ring-enhanced IDH-wildtype gliomas with necrosis contain few normal cells, non-contrast-enhanced IDH-wildtype gliomas often show a higher N-acetylaspartate peak on MR spectroscopy than IDH-mutant gliomas [20]. IDH-wildtype gliomas exhibit greater infiltrative potential at the tumor margin than at the necrotic core [21]. In addition, invasion-related genes are upregulated in regions with low tumor cell density [22]. Therefore, in non-contrast-enhanced IDH-wildtype gliomas, which likely represent an earlier stage without necrosis, the tumor grows in an infiltrative rather than compressive manner. Central necrosis is another potential cause of reduced FDG uptake in glioblastoma. However, non-contrast-enhanced gliomas generally contain little central necrosis, and none of the tumors in the present cohort demonstrated pseudopalisading necrosis [23]. Therefore, the contribution of necrosis to the observed hypometabolism is likely minimal. Taken together, these findings may account for the relatively mild hypometabolism observed in non-contrast-enhanced IDH-wildtype gliomas, reflecting the coexistence of normal brain cells.

Another factor influencing FDG uptake is the intrinsic glucose metabolism of tumor cells. In IDH-wildtype gliomas, glycolytic activity tends to be relatively high, reflecting greater malignancy and rapid proliferation. Moreover, nucleotide synthesis in IDH-wildtype gliomas is heavily dependent on glucose metabolism [5]. In contrast, IDH-mutant tumors primarily depend on the salvage pathway, with little reliance on glucose metabolism. This metabolic shift is accompanied by a reduced expression of key glycolytic enzymes, including hexokinase 1 (HK1) and pyruvate kinase M2 (PKM2) [24,25].

Accordingly, the tumor-associated hypometabolic regions identified on FDG PET in the present study likely reflect the net effect of these opposing influences. While increased glycolysis in tumor components may partially preserve or increase FDG uptake, the loss, displacement, or reduced density of normal brain cells with high physiological glucose metabolism may decrease it. This integrated effect may contribute to the distinct patterns observed between IDH-wildtype and IDH-mutant gliomas.

In our cohort, IDH-wildtype gliomas tended to show a broad area of mildly decreased FDG uptake (Z2–3), which may reflect the partial offsetting of tumor-related reduction in physiological uptake by relatively preserved tumor glycolytic activity. In contrast, IDH-mutant gliomas more often showed extensive areas of markedly decreased uptake (Z6–), which may reflect lower tumor glycolytic activity, together with a greater contribution from the loss or displacement of surrounding normal brain tissue. Therefore, applying a color scale focused on the Z-score range from −2 to −6 effectively highlights differences by IDH status. In the present visual assessment, 20 of 26 IDH-wildtype gliomas (76.9%) were classified into the narrowest Z6– category, compared with 20 of 85 IDH-mutant gliomas (23.5%), supporting the utility of this color scale.

In addition to the distribution of tumor-associated hypometabolic regions, we analyzed their morphological characteristics and sphericity. In IDH-wildtype gliomas, the hypometabolic areas tended to appear more irregular and less spherical. This finding likely reflects their characteristic infiltrative growth along the perivascular spaces and white matter tracts, extending into the surrounding brain tissue [22]. Previous MRI studies have also reported irregular tumor margins and shapes in IDH-wildtype gliomas [18], and our FDG PET-based findings are consistent with these observations. Very low sphericity was observed in 15 of 26 IDH-wildtype gliomas (57.7%) but in only 14 of 85 IDH-mutant gliomas (16.5%), indicating that sphericity was well reflected in the visual assessment of IDH status.

In this study, the visual classification guided by representative reference cases correlated with the quantitative parameters. The interobserver agreement for the four-point visual ratings was high for Z6– extent and moderate to substantial for sphericity. These findings indicate that visual assessment can achieve moderate-to-high interobserver reproducibility, depending on the feature being assessed. Subdividing continuous findings into four ordinal categories may be challenging without representative reference cases. However, for IDH status estimation in clinical practice, distinguishing grade 1 (i.e., the narrowest Z6– extent or very low sphericity) from grades 2–4 may be more relevant than the strict discrimination among all categories. This may allow for a simplified visual interpretation.

Under the 2021 WHO classification, IDH status is central to the classification of adult-type diffuse gliomas. IDH-wildtype tumors generally have a worse prognosis than IDH-mutant tumors but may be difficult to distinguish in non-contrast-enhanced gliomas. Non-contrast-enhanced IDH-wildtype gliomas are increasingly recognized as a clinically important subgroup because their non-contrast-enhanced tumor regions often harbor infiltrating tumor cells [9,10]. Several imaging biomarkers have been proposed for predicting IDH status in non-contrast-enhanced gliomas. MRI biomarkers, such as the T2/FLAIR mismatch sign, arterial spin labeling, diffusion-weighted imaging, and perfusion MRI, as well as amino acid PET, have demonstrated diagnostic utility [26,27,28,29,30]. Recently, combinations of multiple MRI biomarkers or MRI with amino acid PET have been investigated to further improve diagnostic performance [26,28,29,30]. In contrast, the present study investigated the prediction of IDH status using widely available FDG PET alone. To our knowledge, this is the first study to apply a voxel-wise Z-score analysis of FDG PET in gliomas. Rather than relying on absolute FDG uptake, this approach characterizes the spatial distribution and severity of tumor-associated hypometabolism, thereby providing complementary information on tumor biology. Although FDG PET is generally considered to have limited utility in glioma evaluation because FDG uptake is often inconspicuous [2,7], our findings suggest that this approach may provide clinically useful information for estimating IDH status in non-contrast-enhanced gliomas. For broader clinical implementation, voxel-wise Z-score analysis requires an appropriate normal FDG PET database acquired under comparable imaging conditions. Constructing such a database may be challenging for individual institutions. However, publicly available reference databases and image harmonization techniques may provide practical alternatives for implementing this approach [31,32].

In the brain, FDG hypometabolism reflects reduced neuronal activity and is a well-established diagnostic marker for dementia syndromes, epilepsy, and subtle brain injuries [33,34,35]. In temporal lobe epilepsy, FDG hypometabolism has also been incorporated into surgical planning [36], and decreased FDG uptake in the resected region is associated with preserved cognitive function after surgery [37]. Similarly, in gliomas, areas of hypometabolism may represent regions in which normal brain function is lost, potentially serving as a practical guide for safe maximal resection.

The extent of the tumor remains an important clinical consideration. A decrease in the Z-score likely reflects a reduction in normal neurons, as well as infiltration or compression caused by the proliferation of low-grade glioma cells [24]. In our study, the Z-tumor volume exceeded the T2 high-intensity region, and visual comparison showed general concordance between the VOI-T2 and Z-tumor VOIs. However, some regions within the T2 hyperintense area showed no decrease in FDG uptake, whereas some hypometabolic regions showed no T2 hyperintensity. These findings suggest that FDG hypometabolism can provide information complementary to structural MRI in the evaluation of tumor extent. Previous studies have reported that tumor cell infiltration may extend beyond the T2 hyperintense region on MRI [38,39]. However, the pathological significance of these discordant regions remains unclear. Further studies integrating FDG PET, MRI, and histopathological findings are needed to better define the tumor extent and clinical significance of hypometabolic abnormalities. In the context of increasing interest in supratotal resection strategies, such as FLAIRectomy [40], the potential role of FDG hypometabolism beyond MRI abnormalities as a complementary marker of tumor extent warrants further study.

This study has some limitations. First, this was a retrospective, single-center study, and external validation was not performed. In addition, only 26 patients had IDH-wildtype gliomas. Although the inter-reader reproducibility of tumor-associated hypometabolic region delineation was confirmed with a high Dice similarity coefficient, all evaluations were performed within the same cohort. Therefore, further validation in larger independent cohorts is required before broad clinical application of these findings. Second, a small number of patients (*n* = 3) were older than the age range covered by the normal database (≤69 years). However, excluding these patients did not materially change the results, and the associations of the Z6– proportion and sphericity with IDH status remained significant. Third, no established Z-score thresholds currently exist for defining tumor-associated hypometabolism. Although Z < −2 was predefined based on previous voxel-based brain PET studies, the Z6– threshold was selected as an exploratory cutoff for visual assessment. Because both tumor delineation and hypometabolic volume depend on the selected Z-score thresholds, further studies are required to establish standardized criteria. Fourth, because the Z-tumor was defined solely as the hypometabolic region (Z < −2), tumors with preserved or increased FDG uptake may have been underestimated. Accordingly, this method is most applicable to non-contrast-enhanced gliomas with predominant hypometabolism, whereas its applicability to contrast-enhanced gliomas, in which contrast enhancement and central necrosis may substantially influence FDG uptake, remains to be determined. Finally, because tumor grade and IDH status are intrinsically linked in the WHO 2021 classification [8], the present study could not determine whether the observed hypometabolic patterns reflected IDH biology, tumor grade, or both.

## 5. Conclusions

Z-score analysis enabled both visual assessment and quantitative evaluation of non-contrast-enhanced gliomas on FDG PET. Conventional FDG PET assessment primarily focuses on increased FDG uptake, whereas our approach introduces a novel method for evaluating tumor-associated hypometabolic regions. Using this approach, we identified characteristic patterns of FDG hypometabolism across glioma subtypes according to the WHO 2021 classification. A greater proportion of severe tumor-associated hypometabolic regions and higher sphericity were characteristic of IDH-mutant gliomas. The combination of these visually recognizable features may provide useful imaging biomarkers for estimating IDH status in non-contrast-enhanced gliomas. Our findings expand the clinical utility of FDG PET and provide a new framework for metabolic evaluation of non-contrast-enhanced gliomas. Further prospective multicenter studies are warranted to validate these findings and establish the clinical utility of this approach.

## Figures and Tables

**Table 1:** Baseline characteristics and imaging parameters of the three subtypes.

	ALL (*n* = 116)	G (*n* = 26)	A (*n* = 54)	O (*n* = 36)	*p*-Value
Age (years)	42.6 ± 13.0	54.3 ± 14.8	37.8 ± 11.1	41.3 ± 8.8	<0.001 ***
Sex (male)	60	11	31	18	0.44
WHO grade					<0.001 ***
Grade 2	37	–	11	26	
Grade 3	52	–	42	10	
Grade 4	27	26	1	–	
VOI-T2 volume (mL)	33.7 ± 30.2	28.6 ± 27.7	39.5 ± 35.8	28.7 ± 20.1	0.32
Z-tumor VOI volume (mL)	57.1 ± 52.9	56.2 ± 65.8	64.5 ± 54.2	46.5 ± 37.9	0.23
Z-tumor VOI/VOI-T2 volume	2.11 ± 1.97	2.89 ± 3.28	2.0 ± 1.58	1.71 ± 0.82	0.5
Max T/N ratio	0.68 ± 0.24	0.78 ± 0.21	0.57 ± 0.15	0.77 ± 0.30	<0.001 ***
Min T/N ratio	0.37 ± 0.11	0.42 ± 0.13	0.31 ± 0.09	0.42 ± 0.10	<0.001 ***
Mean Z-score †	−4.4 ± 1.3	−3.5 ± 0.8	−5.0 ± 1.4	−4.1 ± 1.0	<0.001 ***
Min Z-score	−12.7 ± 7.2	−9.2 ± 5.2	−15.4 ± 7.7	−11.1 ± 5.9	<0.001 ***
Sphericity	0.65 ± 0.11	0.60 ± 0.11	0.67 ± 0.11	0.67 ± 0.10	0.009 **

Data are presented as mean ± standard deviation or as counts. G, glioblastoma, isocitrate dehydrogenase (IDH)-wildtype; A, astrocytoma, IDH-mutant; O, oligodendroglioma, IDH-mutant and 1p/19q-codeleted; WHO, World Health Organization; T/N ratio, tumor-to-normal ratio. –, not applicable. ** *p* < 0.01; *** *p* < 0.001. † All pairwise comparisons were significant (Steel–Dwass test).

## Data Availability

The raw data supporting the conclusions of this study will be made available by the authors upon request.

## Supplementary Materials

The following supporting information can be downloaded at https://www.mdpi.com/article/10.3390/cancers18142298/s1, Supplementary Material S1: Univariate and multivariate analyses for predicting IDH status; Supplementary Material S2: Summary of receiver operating characteristic analysis for three predictive models; Supplementary Material S3: Visual guide for Z6– and sphericity evaluation; Supplementary Material S4: Comparison of VOI-T2 and Z-threshold volumes (mL) among the three molecular subtypes.

## Abbreviations

The following abbreviations are used in this manuscript:

FDG PET	^18^F-Fluorodeoxyglucose Positron Emission Tomography
WHO	World Health Organization
IDH	Isocitrate Dehydrogenase
SD	Standard Deviation
G	Glioblastoma, IDH-Wildtype
A	Astrocytoma, IDH-Mutant
O	Oligodendroglioma, IDH-Mutant and 1p/19q-Codeleted
MRI	Magnetic Resonance Imaging
T1WI	T1-Weighted Imaging
T2WI	T2-Weighted Imaging
3D	Three-Dimensional
VOI	Volume of Interest
T/N Ratio	Tumor-to-Normal Ratio
SUV	Standardized Uptake Value
eZIS	Easy Z-Score Imaging System
ROC	Receiver Operating Characteristic
AUC	Area Under the Curve
HK1	Hexokinase 1
PKM2	Pyruvate Kinase M2
