# Supplementary material for: Glucose Hypometabolic Pattern and Prediction of Isocitrate Dehydrogenase Status in Non-Contrast-Enhanced Glioma Using Z-Score-Based 18F-Fluorodeoxyglucose Positron Emission Tomography
Source: Cancers (Basel). 2026 Jul 17;18(14):2298. doi: 10.3390/cancers18142298 (PMC13406570; doi:10.3390/cancers18142298)
Supplement: Supplementary file 1 [file cancers-18-02298-s001.zip › cancers-4412939-supplementary.pdf]

Supplementary Material S1: Univariate and multivariate analyses for predicting IDH status

	IDH-wildtype	IDH-mutant	Univariate	Multivariate	
			***p*-value**	**Odds ratio (95% CI)**	***p*-value**
Z6–	7.1 ± 9.5	21.9 ± 16.6	<0.001 ***	1.089 (1.046–1.145)	<0.001 ***
Sphericity	0.6 ± 0.11	0.67 ± 0.1	0.003**	1240.6 (16.6–175,881)	0.002 **

Values are presented as mean ± standard deviation. CI: confidence interval; IDH: isocitrate dehydrogenase. Univariate analyses were performed using the Mann–Whitney U test. Multivariate analysis was conducted using a logistic regression model. Odds ratios are shown with 95% CI.

$^{**}p < 0.01$; $^{***}p < 0.001$

Supplementary Material S2. Summary of receiver operating characteristic analysis for three predictive models

**Model**	**AUC (95% CI)**	**Cutoff**	**Sensitivity (%)**	**Specificity (%)**	**TP**	**FP**	**FN**	**TN**
Z6–	0.755 (0.661–0.849)	10.8	68.9	80.8	62	5	28	21
Sphericity	0.694 (0.575–0.813)	0.63	70	73.1	63	7	27	19
Combined model	0.839 (0.760–0.919)	–	78.9	76.9	71	6	19	20

Sensitivity, specificity, true positives (TP), false positives (FP), false negatives (FN), and true negatives (TN) were calculated using the optimal cutoff derived from Youden's index. The combined model incorporating Z6– and sphericity was generated using multivariable logistic regression. The Area Under the Curve (AUC) of the combined model was significantly higher than that of Z6– (p = 0.012) and sphericity (p = 0.001).

Supplementary Material S3. Visual guide for Z6– and sphericity evaluation

Supplementary Material S3a. Z6– (proportion of black voxels)

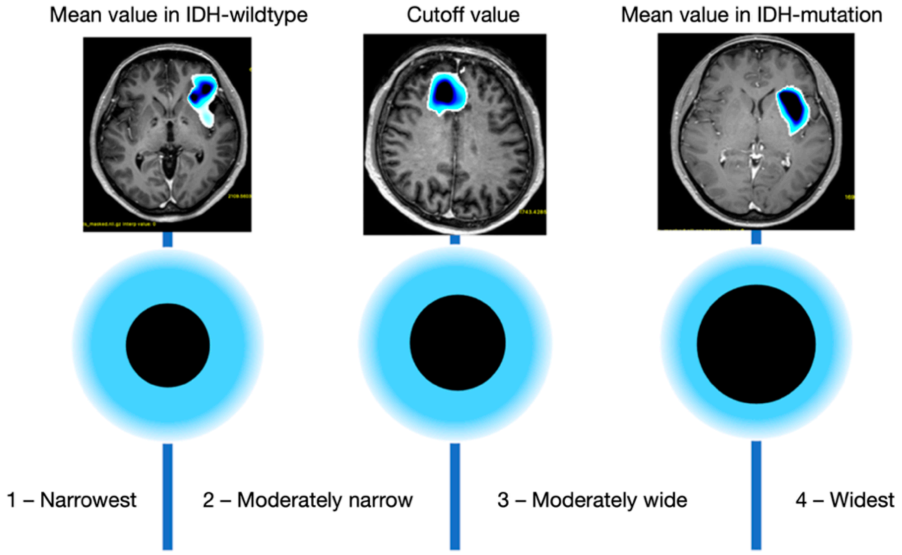

Supplementary Material S3b. Sphericity

Sphericity increases as the shape approaches a sphere and decreases as it becomes more irregular or elongated.

$$= \frac{\pi^{1/3}(6V)^{2/3}}{A}$$

V: volume
A: surface area

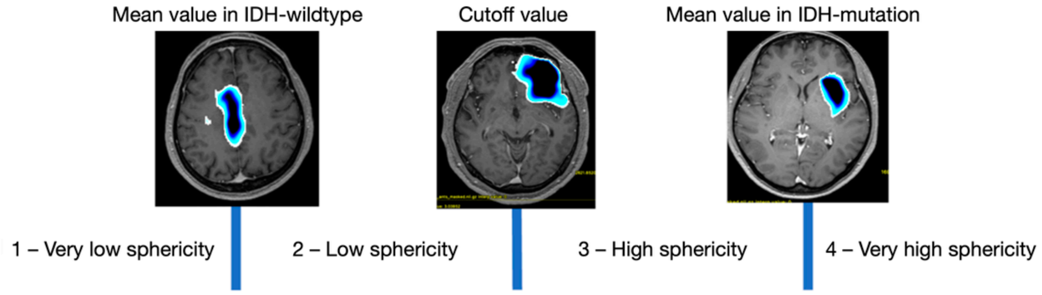

Supplementary Material S3c. Boxplots of visual assessments and quantitative values.

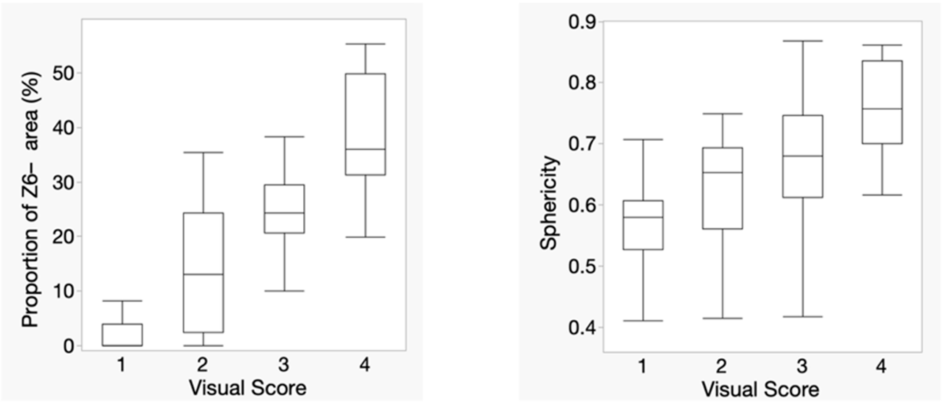

Supplementary Material S4. Comparison of VOI-T2 and Z-threshold volumes (mL) among the three molecular subtypes

	**ALL (*n* = 116)**	***p*-Value†**	**G (*n* = 37)**	**A (*n* = 54)**	**O (*n* = 25)**	***p*-Value‡**
VOI-T2	33.7 ± 30.2	–	28.6 ± 27.7	39.5 ± 35.8	28.7 ± 20.1	0.322
Z < −2	57.1 ± 52.9	<0.001 ***	56.2 ± 65.8	64.5 ± 54.2	46.5 ± 37.9	0.228
Z < −3	39.3 ± 40.8	0.006 **	34.5 ± 44.9	47.4 ± 45.1	30.6 ± 27.3	0.072
Z < −4	28.2 ± 32.3	<0.001 ***	21.5 ± 30.3	36.4 ± 37.8	20.7 ± 20.4	0.016 *
Z < −5	20.5 ± 25.8	<0.001 ***	12.6 ± 18.5	28.5 ± 31.6	14.3 ± 15.7	0.005 **
Z < −6	15.1 ± 21.1	<0.001 ***	6.7 ± 11.4	22.5 ± 26.5	9.9 ± 11.8	<0.001 ***
Z < −7	11.2 ± 17.3	<0.001 ***	3.9 ± 7.6	17.8 ± 22.1	6.8 ± 8.9	<0.001 ***
Z < −8	8.4 ± 14.0	<0.001 ***	2.3 ± 5.1	13.9 ± 18.1	4.6 ± 6.7	<0.001 ***
Z < −9	6.2 ± 11.1	<0.001 ***	1.4 ± 3.5	10.7 ± 14.4	3.1 ± 5.0	<0.001 ***
Z < −10	4.6 ± 8.7	<0.001 ***	0.9 ± 2.4	8.1 ± 11.4	2.1 ± 3.9	<0.001 ***

Data are expressed as mean ± standard deviation (mL).

† *p*-values from paired comparisons between each Z-threshold volume and VOI T2 volume. ‡ *p*-values from Kruskal–Wallis tests among the three molecular subtypes. **p* < 0.05, ***p* < 0.01, ****p* < 0.001.
